# Efficacy of post-operative partial weight-bearing after total knee arthroplasty – a prospective observational trial

**DOI:** 10.1007/s00264-023-05783-0

**Published:** 2023-03-24

**Authors:** Philip Mark Anderson, Tizian Heinz, Elena Scholmann, Annette Eidmann, Jörg Arnholdt, Maximilian Rudert, Boris Michael Holzapfel, Ioannis Stratos

**Affiliations:** 1 Department of Orthopedics at Koenig-Ludwig-Haus, University of Wuerzburg, Brettreichstraße 11, 97074 Würzburg, Germany; 2 Department of Orthopedics and Trauma Surgery, Musculoskeletal University Center Munich (MUM), University Hospital, LMU Munich, Marchioninistr. 15, 81377 Munich, Germany

**Keywords:** Partial weight-bearing, Total knee arthroplasty, Force sensor, Patient compliance

## Abstract

**Purpose:**

There is little evidence proving the concept of partial weight-bearing to be efficient and feasible. Using insole pressure measurement systems, this study aimed to explore the compliance to prescribed weight-bearing restrictions after total knee arthroplasty (TKA).

**Methods:**

50 patients after TKA were recruited in a prospective manner. They were advised to limit weight-bearing of the affected limb to 200 N. True load was measured via insole force-sensors on day one after surgery (M1) and before discharge (M2). Compliance to the rehabilitation protocol was the primary outcome parameter.

**Results:**

At M1 and M2 compliance to the rehabilitation protocol was 0% und 2%, respectively. 84% (M1) and 90% (M2) of patients overloaded the affected limb during every step. The affected limb was loaded with 50% ± 14% (M1) and 57% ± 17% (M2) of body weight. Patients older than 65 loaded the affected limb on average 17% (M1) and 34% (M2) more than their younger counterparts did. This difference was even more pronounced when walking stairs up (49% increase on average) and down (53% increase on average).

**Conclusion:**

Surgeons must take into consideration that the ability to maintain partial weight-bearing after TKA is highly dependent on the age of the patient and the achievable load reduction is determined by the patient’s body weight.

## Introduction

Partial weight-bearing after lower limb surgery has a long tradition aimed at preventing the patient from overloading the affected limb during tissue healing. While partial weight-bearing has been widely prescribed by orthopaedic and trauma surgeons, there is little evidence proving this concept to be efficient and feasible for the majority of patients. Furthermore, the possible negative effects of immobility and reduced activity have been well described, e. g. muscle hypotrophy, venous thromboembolism and loss of function and autonomy, especially in elderly patients. Taking this into account, the concept of partial weight-bearing has been questioned over the last years [1]. In addition—and especially in total hip and knee arthroplasty (TJA)—so called “fast-track concepts” have evolved, which are intended to optimize the rehabilitation after TJA [2]. Besides many other components, early mobilization with full weight-bearing is an essential part of these widely adopted programs.

Simultaneously to the evolution of the rehabilitation concepts after TJA, real-time measurement of patients’ kinetic parameters after surgery has become widely available using wireless wearable sensors, e. g. insole pressure measurement systems that calculate the ground reaction forces (GRF) during gait. This procedure offers the chance to investigate the feasibility and necessity of partial weight-bearing concepts during the standardized rehabilitation under everyday conditions. The insole pressure measurement devices have been used in the past to elucidate movement pattern biofeedback training after total knee arthroplasty [3]. Moreover, instrumented insoles are accepted devices for real-time feedback during physiotherapy in order to improve patient outcomes [4, 5]. Thus instrumented insoles are widely accepted in orthopaedic surgery and sports medicine [6].

This study was designed to explore the compliance to the prescribed weight-bearing restrictions after total knee arthroplasty (TKA) at a high-volume academic medical centre. The main hypothesis was that limiting the load on the affected limb to 200 Newton would not be feasible for the majority of patients. Furthermore, it was intended to explore if the amount of load on the affected limb correlates to the intensity of pain during early mobilization after TKA.

## Material and methods

Patients older than 18 years undergoing primary total knee arthroplasty for osteoarthritis were recruited in a prospective manner beginning in July 2020. The following exclusion criteria were defined:

- Dementia or other cognitive impairments leading to non-compliance
- Immobility related to diseases of the hip or the contralateral leg making partial weight-bearing impossible
- Use of revision implants
- Complications during surgery leading to a change in the standardized rehabilitation protocol

The standardized rehabilitation started the day after surgery. Patients were informed and instructed by physiotherapists. They were advised to limit the weight-bearing of the affected limb to 20 kilograms (kg), equaling 200 Newton (N) weight force after rounding up gravity acceleration from 9.81 to 10 N/kg. Crutches were used for ambulation.

After surgery, the true weight-bearing during training with the physiotherapists was measured twice. First, on day one or two after surgery (M1), and second on the day before discharge (M2). At M1 the GRF and the number of steps during gait were measured in a hallway covering a standardized distance of 50 m. At M2, in addition to the same measurement as in M1, the GRF were also measured going up and down ten steps in a stairway. Patients were not informed about their performance in order to avoid influencing on their behavior. After training, the patients were asked to estimate their compliance to the advised weight-bearing by rating on a Likert scale from 0 (“not at all”) to 3 (“perfectly”) and to rate the severity of pain during training on a numerical rating scale (NRS) from 0 (“no pain”) to 10 (“worst pain imaginable”).

The GRF were measured by wearable size-adjusted insole force-sensors (Loadsol, novel GmbH, Germany) after calibrating each insole using the known body weight. These highly accurate insoles estimate the GRF by measuring the force at the plantar surface of the foot using capacitor-based sensors [7, 8]. To avoid the influence of various shoe styles on the GRF, the same type of shoes were used by all patients, differing only in size (Striker casual shoe, JAKO AG, Germany). Data were sent via wireless transmission to a portable device (iPad, Apple, USA). For further evaluation, the data were transmitted to a personal computer as plain text data files and analyzed using a self-programmed Visual-Basic algorithm and Excel 365 (Microsoft Corporation, USA).

Besides this data, the following information was gathered for each patient:

- Weight
- Gender
- ASA Score
- Age
- Number of relevant comorbidities
- Length of stay (LOS)
- Orthopaedic complications within 90 days

Furthermore, patients were contacted three months after surgery and asked to rate their subjective limitation during rehabilitation caused by the prescribed weight-bearing restriction. A Likert scale was used, with ratings from 0 (“no limitation”) to 3 (“very strong limitation”).

The primary outcome parameter was the compliance to the prescribed partial weight-bearing protocol. Compliance was defined as a rate of less than 10% of steps during M1 and M2 in which the affected limb was overloaded (> 200 N). The concept of partial weight-bearing with 200 N was defined as “rational” if more than 80% of the patients were compliant, “doubtful” if 50–80% were compliant and “senseless” if less than 50% were compliant.

The following secondary outcome parameters were defined:

- Average maximum load of the affected limb (maximum load per step divided by the number of steps) during M1 and M2
- Ratio of load on the affected limb to body weight force
- Weight-bearing reduction of the affected limb in comparison to the unaffected one
- Correlation between the load of the affected limb and the pain during ambulation
- Correlation between the true load of the affected limb and the estimated load by the patient
- Correlation between the patient’s weight and the maximum load
- Extent of subjective limitation during rehabilitation caused by limited weight-bearing
- Rate of orthopaedic complications within 90 days
- Subgroup analysis of patients older and younger than 65 years

A pre-hoc power calculation was performed and required 30–100 patients, depending on the estimated percentage of patients being compliant. For this, it was planned to begin with 50 patients.

Data analysis was conducted by Jamovi v. 2.0.0. Continuous data are shown as mean ± standard deviation. Ordinal data are shown as median with the 25% and 75% percentile. Categorial data are shown as rates in percent.

An unpaired t-test was used to compare mean load values between two independent groups. A paired t-test analysis was used to analyze differences between two variables for the same subject (in the present analysis: operated extremity vs. contralateral extremity). In case of a failed normality test groups were compared by non-parametric rank sum test (Mann–Whitney) or an ANOVA on ranks (Kruskal–Wallis) for comparison of more than two groups. Linear regression analyses were chosen to show correlations between weight and plantar force. An F-Test was used to calculate a statistical difference between the slope of a regression line and a zero-slope. Correlation analysis between pain and load was conducted with the Spearman Correlation. The significance level was set at *p* < 0.05. No correction of significance level was implemented for multiple comparisons in the secondary outcome parameters.

## Results

Patient recruitment stopped after including 50 patients of whom 33 were female (66%). As exclusion criteria was fully available before recruitment, no dropouts occurred. The average age at time of surgery was 66 ± nine years. 28 left knees (56%) were operated on. In 40 patients (80%) a cruciate-retaining implant was used, whereas in the remaining ten patients (20%) a cruciate-substituting implant was chosen. The median ASA Score was 2 (ASA 1: 12%; ASA 2: 64%; ASA 3: 18%; ASA 4: 2%). The average LOS was 6 ± 1 days.

**Fig. 1:**
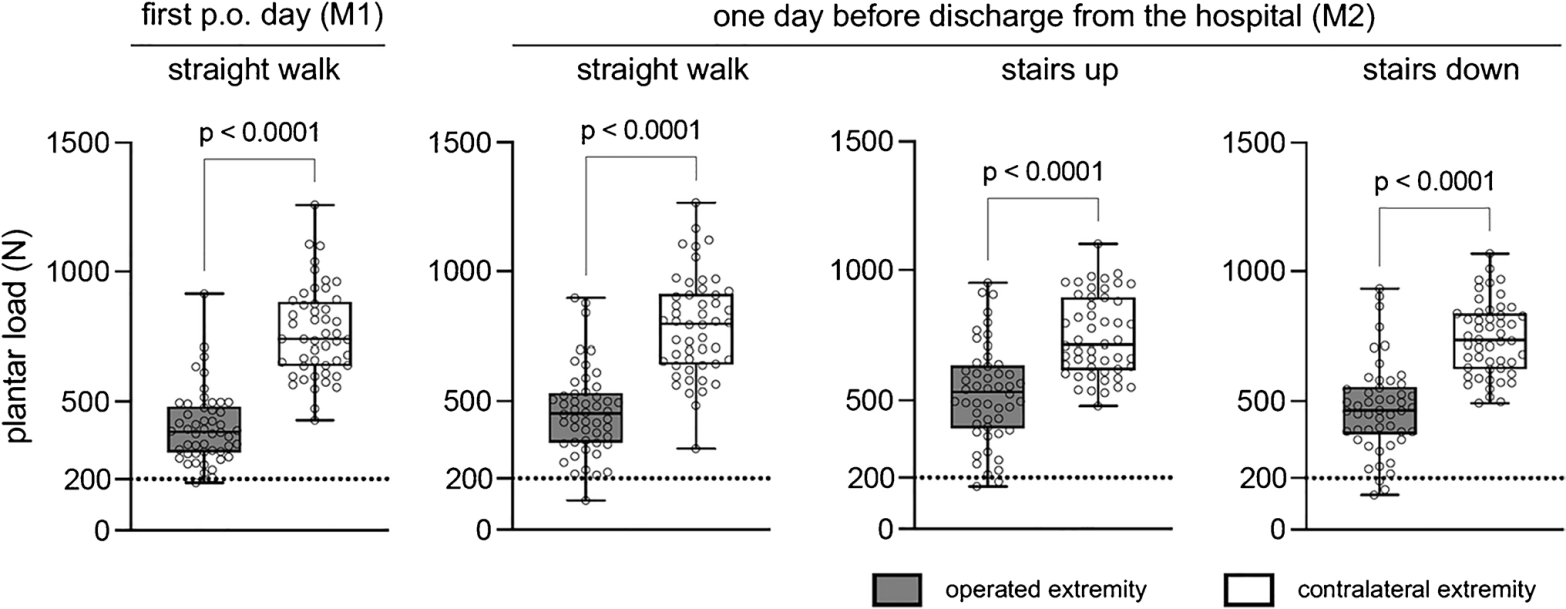
Plantar load (in N) on the operated limb (gray boxplots) and on the contralateral limb (white boxplots) after TKA. All patients were instructed to partially weight-bear with 200 N post-operatively. Analysis was performed on the first day and one day before hospital discharge. Paired t-test analysis for values of the operated extremity and the contralateral extremity. The exact level of statistical significance is shown in the graph

**Fig. 2:**
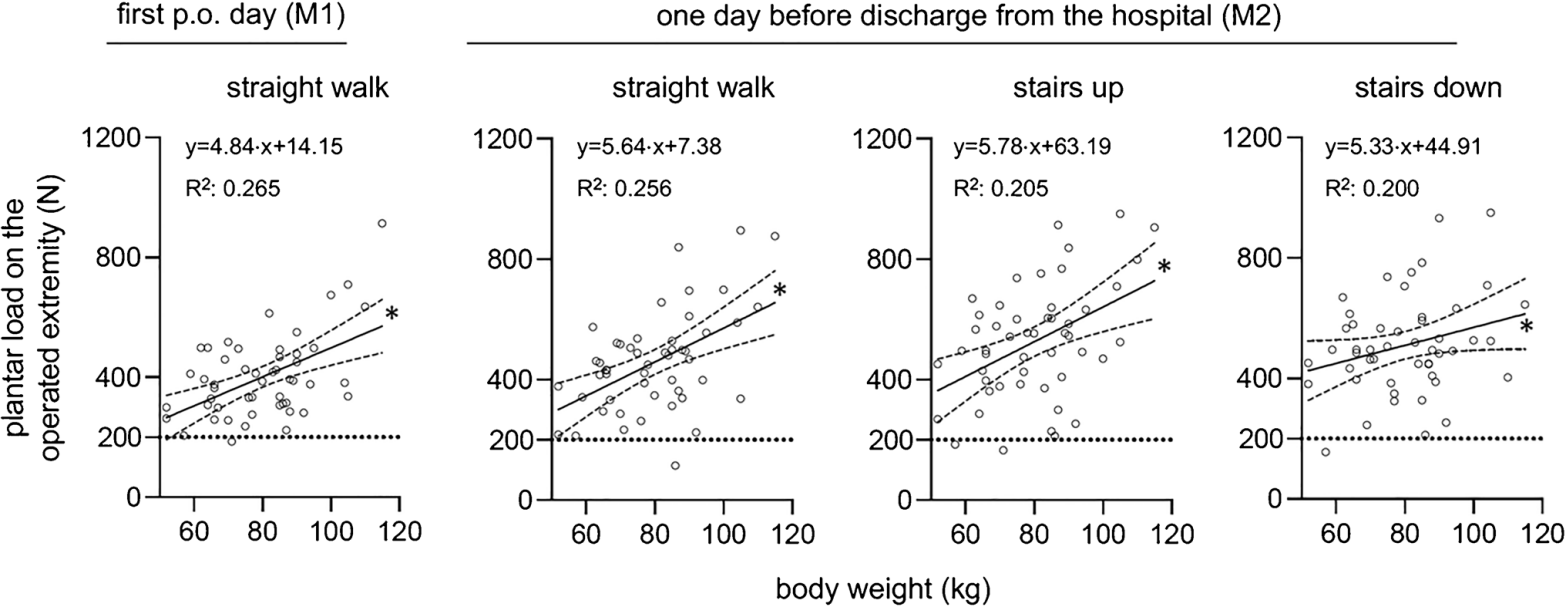
Linear regression of the plantar load on the operated limb (in N) after TKA and the patient’s body weight (in kg). All patients were instructed to partially weight-bear with 200 N post-operatively. Each diagram shows the regression line, the 95% coefficient interval as well as the regression equation and the coefficient of determination (R2). The * shows that the slope of each curve is significantly non-zero (F-test; *p* < 0.01)

First day post-operative (M1): At M1 no patient was compliant to the limited weight-bearing protocol of 200 N. 42 patients (84%) overloaded the affected limb in every step they took during measurement. The mean number of steps was 40 ± 11 and the mean maximum load during each step was 401 ± 140 N for the affected and 765 ± 174 N for the contralateral limb (*p* < 0.05) (Fig. 1). On average, the affected limb was loaded with half of the body weight (50% ± 14%). The median pain during ambulation at M1 was rated as 4 (3—5). There was no significant correlation (*p* = 0.342) between pain and the mean maximum load of the affected limb. The self-estimated compliance to the restricted weight-bearing protocol showed no significant correlation to the mean maximum load (p = 0.443). The patients’ weight was positively correlated to the mean maximum load of the affected limb (*p* < 0.05) (Fig. 2).

One day before discharge from the hospital (M2): At M2 only one patient (2%) was compliant during measurement in the hallway and down the stairs, but not up the stairs. 45 patients (90%) overloaded the affected limb in every step they took in the hallway and 46 patients (92%) while climbing and descending stairs. The mean number of steps in the hallway were 56 ± 17 and the mean maximum load during each step was 458 ± 161 N in the hallway, 525 ± 190 N up the stairs and 471 ± 178 N down the stairs. For the unaffected limb the mean maximum loads were significantly higher (*p* < 0.05 for all groups): 789 ± 194 N, 745 ± 155 N and 740 ± 142 N, respectively (Fig. 1). On average, the affected limb was loaded with over half of the body weight during all three measurements: 57% ± 17%, 66% ± 21% and 59% ± 20%. The median pain during ambulation at M2 was rated 3 (2—3) in the hallway, 3 (1—4) down and 3 (1—4) up the stairs. There was no significant correlation between pain and the mean maximum load of the affected limb during ambulation in the hallway or up or down the stairs (*p* > 0.05 for all groups). The self-estimated compliance to the restricted weight-bearing protocol showed no significant correlation to the mean maximum load (*p* = 0.630).

The patients’ weight was positively correlated to the mean maximum load of the affected limb in the hallway (*p* < 0.05) as well as up (*p* < 0.05) and down the stairs (*p* < 0.05) (Fig. 2). When comparing the number of steps and the mean maximum load during measurement in the hallway at M1 and M2, there was a statistically significant rise in the number of steps (*p* < 0.05) and in the mean maximum load (*p* < 0.05). When patients were called three months after surgery, the median subjective limitation during rehabilitation caused by the restricted weight-bearing was rated 1 (0—1). There was no significant correlation (*p* = 0.489) between the extent of subjective limitation and the mean maximum load at M2. Within the first three months after surgery there were no cases of periprosthetic fractures, two cases (4%) of hematoma, one case (2%) of rupture of the extensor mechanism and no cases of periprosthetic joint infections.

Subgroup analysis patients ≤ 65 years vs. patients > 65 years.

**Fig. 3:**
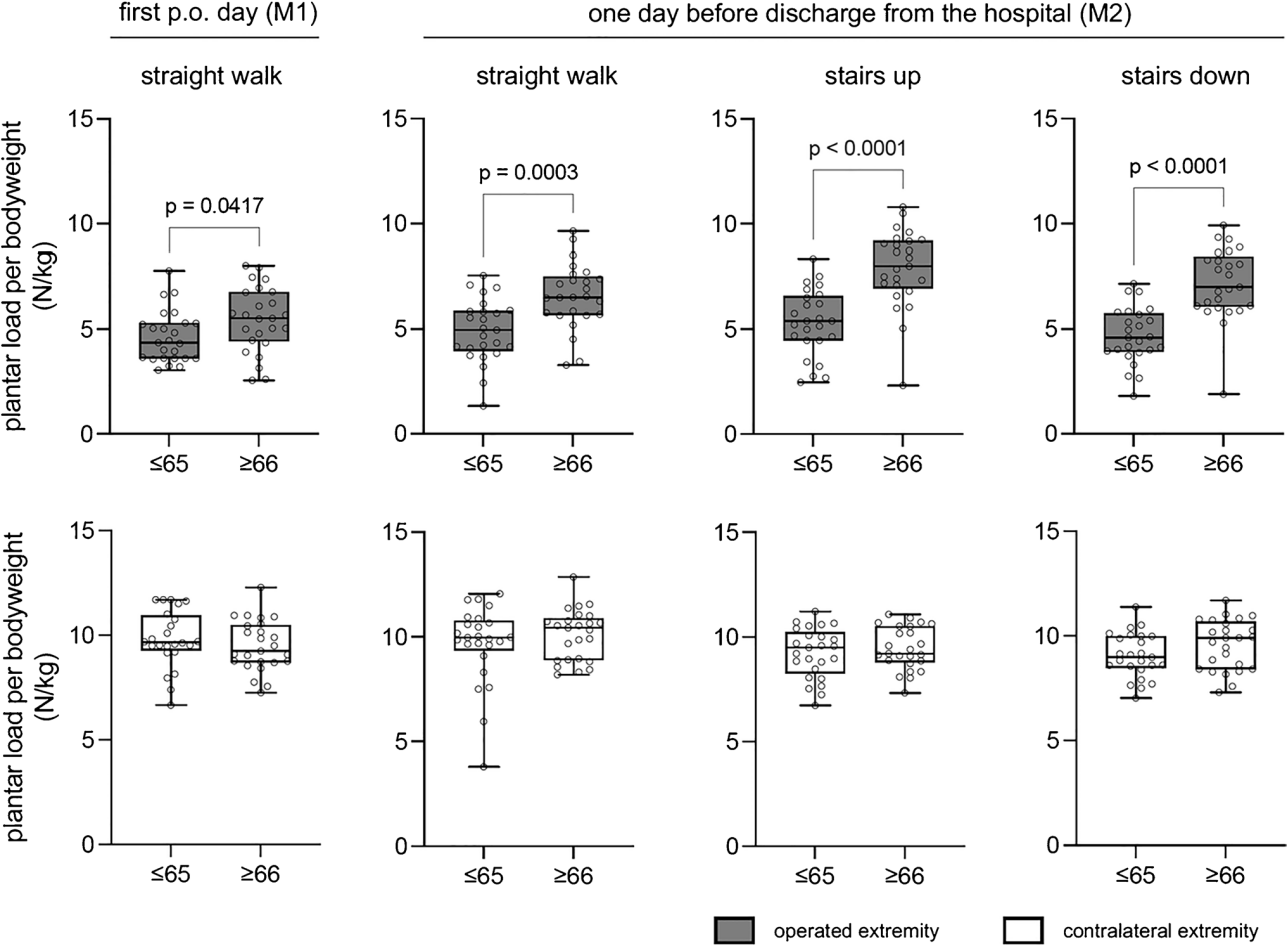
Plantar load per body weight (N/kg) after TKA on the operated limb (gray boxplots; upper panel) and on the contralateral limb (white boxplots; lower panel). All patients were instructed to partially weight-bear with 200 N post-operatively. Subgroup analysis was performed for patients ≤ 65 (*n* = 25) and > 65 years (*n* = 25) of age. The exact level of statistical significance is shown in the graph when *p* < 0.05

At the time of surgery, 25 patients were 65 years or younger and 25 patients were older than 65 years. These groups differed only in the mean LOS (Table 1): patients older than 65 years stayed on average 0.7 days longer than younger patients. The quotient between plantar load (N) and body weight (kg) was used to determine whether younger patients were more capable of maintaining a partial weight-bearing during gait. For the non-operated extremity there were no differences between M1 and M2. Mean values ranged between 9.1 N/kg and 10.1 N/kg in both groups, reflecting full weight-bearing (Fig. 3; lower panel). Significant differences of load were observed for the operated limb between patients ≤ 65 compared to patients > 65 for all measurements (Fig. 3; upper panel). During straight walk at M1 and M2 patients ≤ 65 years showed an average ratio of 4.6 N/kg and 5.3 N/kg, whereas patients > 65 years showed an average ratio of 5.5 N/kg and 7.9 N/kg, reflecting a significant average increase for the older patients of 17% and 34%, respectively (Fig. 3; upper panel). The differences between patients ≤ 65 and > 65 years were even more pronounced at M2 when walking stairs up (≤ 65 years: 5.3 N/kg; > 65 years: 7.9 N/kg; 49% increase on average) and stairs down (≤ 65 years: 4.7 N/kg; > 65 years: 7.2 N/kg; 53% increase on average).

**Table 1:** Subgroup analysis: The factors length of hospital stay, age, weight, ASA-score and pain were analyzed for the subgroups of age (≤ 65 years and > 65 years) (*p* < 0.05)

	Age	
	≤ 65 years (*n* = 25)	> 65 years (*n* = 25)
Length of hospital stay (d)	5.5 ± 1.4	6.2 ± 1.0 ^#^
Age (y)	58.9 ± 5.2	73.9 ± 6.0 ^#^
Weight (kg)	82.6 ± 14.0	77.3 ± 15.6
ASA-score	2 (2–2)	2 (2–2)
Pain scale (1–10)		
First. p.o. day—straight walk	4 (3–5)	4 (3–5)
Day before discharge—straight walk	3 (2–3)	3 (2–3)
Day before discharge—stairs up	3 (1–3)	3 (1–4)
Day before discharge—stairs down	3 (1–3)	3 (1–4)

## Discussion

The most important finding of this study is that patients after TKA are not able to reduce weight-bearing of the affected limb to 200 N and that they are not good at judging their performance. Furthermore, their compliance even deteriorates with time after surgery, indicating that daily training with the physiotherapists does not improve the adherence to the limited weight-bearing protocol. While the concept of partial weight-bearing with 200 N after TKA must therefore be considered as “senseless” (as defined in the introduction), at least it did not lead to severe subjective limitation during rehabilitation.

Despite their inability to adhere to the prescribed partial weight-bearing of 200 N, patients significantly reduced the load of the affected limb in relation to the unaffected one in all measurements. In this manner, the achieved load was highly correlated to the patient’s weight. On average, the operated limb was loaded with approximately half of the body weight force. Thus, crutches enable patients to halve their body weight force on the affected leg. Consequently, surgeons must take the patient’s weight into account when considering partial weight-bearing after TKA. 200 N is a realistic goal only with a patient weighing 40 kg or less.

Next to the patient’s weight, surgeons must take the patient’s age into account. In this study, patients over 65 were much less capable of maintaining partial weight-bearing than patients under 65. Especially on the stairs, the older patients tended to full weight-bearing. This might be explained by the declining mental and physical strength with increasing age, making partial weight-bearing difficult when climbing or descending stairs [9]. It is important to recognize that the study population contained mainly healthy patients (76% ≤ ASA 2). It might be interpreted as “best-case scenario” for partial weight-bearing after surgery of the lower extremity in patients aged 50 years and more. It must therefore be postulated that geriatric multimorbid patients are even less capable of following limited weight-bearing protocols as has been shown for example by Pfeufer et al. in 2019 [10]. In old trauma patients or in case of revision TJA in the elderly, for example, it must therefore be full or no weight-bearing at all [11]. If limited weight-bearing is still prescribed in this population, at least biofeedback methods should be considered as these have shown their ability to improve the patients’ capability to comply to the protocol [12].

Castellarin et al. recently published a study with similar insoles confirming our findings that patients were unable to adhere to limited weight-bearing with 10–20% of the body weight in the first phase after TKA [13]. At day three after surgery only 13% were able to limit weight-bearing to 50% of the body weight without alteration of their gait.

In 2002, Tveit et al. showed that 15 patients after total hip arthroplasty were not able to follow a prescribed limited weight-bearing protocol in a single case and that one third of them was not aware of this [14]. The authors, too, questioned the concept of partial weight-bearing.

Furthermore, the results of this study indicate that partial weight-bearing after TKA is not necessary, as rates of potentially load-associated complications such as fractures were within commonly reported ranges even though nearly all patients were noncompliant with the limited weight-bearing protocol [15]. Concerning this matter, the limited number of recruited patients must be taken into account, as the study was not designed for this outcome parameter and is therefore underpowered. Nevertheless, common practice with widely adopted fast-track concepts including full weight-bearing after TKA has not led to an increase in complications [16]. Generally, there is only limited evidence of the necessity of limited weight-bearing protocols in orthopedic and trauma surgery. Available research mostly shows no benefit of partial weight-bearing [17].

When interpreting the findings of this study, the following limitations must be taken into consideration. First, the quality of the training by the physiotherapists was not validated. Misleading information or insufficient instructions therefore can’t be definitely ruled out. Nevertheless, this seems unlikely as all involved physiotherapists have a long history of supporting patients after TJA, making them very experienced in this field. Secondly, the last measurement was taken before discharge from hospital. Improvement in complying with the limited weight-bearing protocol may have occurred in the subsequent rehabilitation process although this seems highly unlikely since the performance between M1 and M2 deteriorated despite less pain and more training. Finally, this study was limited to patients after TKA, therefore limiting the transfer of the results to limited weight-bearing protocols after other surgical procedures of the lower limb. Nevertheless, TKA is considered one of the most painful procedures in orthopaedic surgery, making it likely that the compliance after less painful procedures might even be worse. Thirdly, possible effects on aseptic loosening of the implants by the initial amount of weight-bearing can’t be ruled out with certainty, but it seems highly unlikely as all implants were cemented and therefore bony ingrowth within the first weeks is not of concern.

## Conclusion

To the best of our knowledge, the present study is the first trial investigating the real load applied to the affected limb after TKA using an insole force sensor in patients trained to maintain partial weight-bearing of 200 N. This rehabilitation protocol is not feasible for the majority of patients after TKA. At best, the load on the affected limb can be reduced to half of the body weight force in younger patients using crutches. Therefore, surgeons must take into consideration that the ability to maintain partial weight-bearing after TKA is highly dependent on the age of the patient and the achievable load reduction is determined by the patient’s body weight.

## Data Availability

Data will be made available by the corresponding author upon reasonable request.
